# Activation of ATP-sensitive potassium channels antagonize nociceptive behavior and hyperexcitability of DRG neurons from rats

**DOI:** 10.1186/1744-8069-7-35

**Published:** 2011-05-14

**Authors:** Xiaona Du, Chao Wang, Hailin Zhang

**Affiliations:** 1 The Key Laboratory of Neural and Vascular Biology, Ministry of Education; The Key Laboratory of New Drug Pharmacology and Toxicology, Hebei Province; Department of Pharmacology, Hebei Medical University, Shijiazhuang, China

**Keywords:** K_ATP_, potassium channels, DRG, excitability, nociception, pinacidil, diazoxide, glyburide

## Abstract

**Background:**

Nociceptive responses to noxious stimuli are initiated at peripheral nociceptor terminals. Ion channels play a vital role in pain signal initiation and conduction. Activation of K_ATP _channels has been implicated in mediating the analgesic effects of agents such as morphine. However, systematic studies regarding the effects of K_ATP _activators on nociception and neuronal excitability are scarce.

**Results:**

In this study, we describe the antagonistic effects of K_ATP _activators pinacidil and diazoxide on nocifensive behavior induced by bradykinin (BK), thermo and mechanical stimuli, and the bradykinin-induced hyperexcitability of DRG neurons. We also found that K_ATP _activators can moderately activate K_ATP _in DRG neurons. Because the effects of K_ATP _activators can be reversed by the K_ATP _blocker glyburide, direct activation of K_ATP _is most likely the underlying mechanism.

**Conclusion:**

This systematic study clearly demonstrates that activation of K_ATP _could have significant modulatory effects on the excitability of sensory neurons and thus on sensory behaviors, such as nociception. K_ATP _activators can be evaluated clinically for the treatment of pain symptoms.

## Background

Ion channels play a vital role in pain signal initiation and conduction [1]. For example, activation of TRPV1 following a heat stimulus generates inward currents in the nociceptor peripheral terminal and results in action potentials in the nociceptor axon, leading to pain sensation [2-5]. The TRP channel family and voltage gated sodium channels are among the most intensively studied ion channels in pain signaling. Until recently, less attention has been paid to the role of potassium (K^+^) channels in pain [6-17]. K^+ ^channels play an essential role in setting the resting membrane potential and in controlling the excitability of neurons. Thus, K^+ ^channels represent potentially attractive peripheral targets for the treatment of pain. One K^+ ^channel that is known to regulate excitability in a variety of central and peripheral neurons is the M channel [18,19]. Activation of the M channel by retigabine inhibits responses to the intrapaw application of carrageenan [7] and bradykinin [6] in rat nociceptive behavioral studies. The M channel blocker XE991 evokes spontaneous pain in rats [12,20].

Another family of K^+ ^channels that has recently been indicated in pain responses is the ATP-sensitive potassium channel (K_ATP_) family. These channels are widely expressed in central neurons, wherein they regulate membrane excitability and neurotransmitter release, and they provide neuroprotection [21,22]. It has been suggested that K_ATP _may mediate the analgesic effects of morphine [23], clonidine [24] and 5-HT1 agonists [25] because the antinociceptive effects of these agents could be reversed by pretreatment with selective K_ATP _antagonists but not other potassium channels blockers [26]. Studies indicate that the nitric oxide (NO) pathway mediates the morphine activation of K_ATP _[16,27]. Indeed, NO donors produce peripheral antinociceptive effects in inflammatory pain [27,28] and directly activate K_ATP _in rat DRG neurons [14].

The existence of K_ATP _in peripheral sensory neurons has only recently been confirmed [8,13-15,17]. However, *in vitro *studies on DRG neurons demonstrate whole-cell K_ATP _currents that either show unusual rectification properties or are expressed in only a subpopulation of the neurons [8,11]. Activation or inhibition of K_ATP _has only minor effects on the resting membrane potential of the subpopulation of neurons [11,15]. It has been suggested [13,15] that K_ATP _may play a major role in large diameter DRG neuron-mediated neuropathic pain. There is also evidence suggesting that the anti-nociceptive effects of K_ATP _activators may stimulate mechanisms that produce anti-nociception through opioid receptor activation [29,30]. However, studies on the effects of direct K_ATP _activation on peripheral antinociception are scarce [31-33].

The primary purpose of the present study is to establish the effects of direct activation of K_ATP _on pain sensation and the excitability of sensory neurons. We performed experiments testing the effects of two K_ATP _activators, diazoxide and pinacidil, on pain behaviors induced by bradykinin and thermal and mechanical stimuli. We also studied the effects of K_ATP _activators on the excitability and whole-cell currents of DRG neurons. The results show that activation of K_ATP _antagonizes the rat nociceptive behavior induced by all tested stimuli and dampens the hyperexcitability of DRG neurons induced by bradykinin.

## Results

### The K_ATP _channel activators antagonize bradykinin-induced spontaneous pain behavior

**Figure 1:**
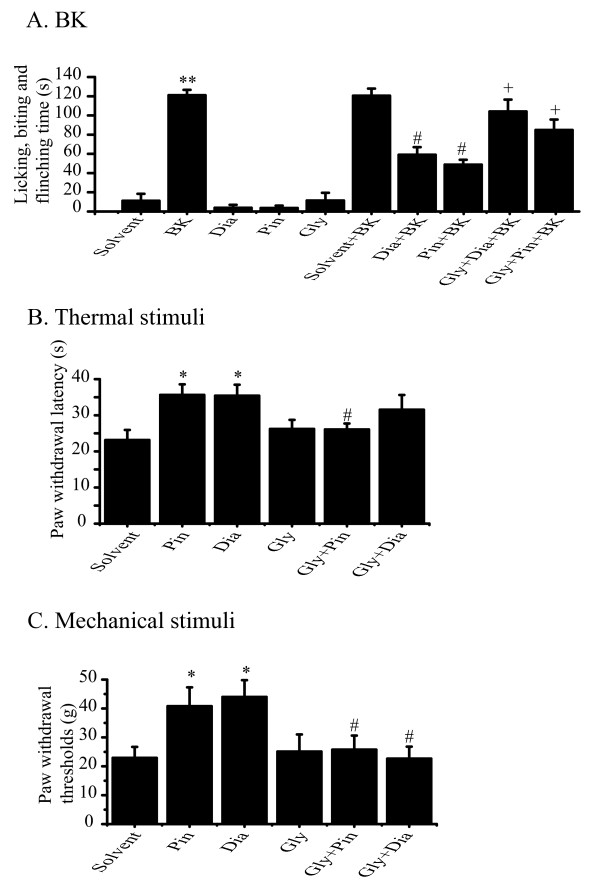
**K_ATP _openers attenuate nocifensive behavior induced by BK, thermal and mechanical stimuli**. (A) Effects of K_ATP _openers on BK-induced nocifensive behavior. For first series of the experiment, solvent (DMSO, 0.5%), BK (200 μM), K_ATP _channel openers pinacidil (Pin, 10 μM), or diazoxide (Dia, 100 μM), or K_ATP _channel blocker glyburide (Gly, 10 μM), were dissolved in saline and injected into the rat hind paw of different rats in a volume of 50 μl in each case. In the second series of the experiments, Pin, Dia, mixture of Pin + Gly, mixture of Dia + Gly or solvent was injected 5 min before the injection of BK plus the mixture (second injection was done into the same site). The time the animals spent licking, biting and flinching the injected paw during 30 min was recorded and shown. n = 8 for each group. **p < 0.01, compared with solvent group; #p < 0.05, compared with BK group; +p < 0.05, compared with BK + Dia or BK + Pin groups. (B) Effects of K_ATP _channel openers on thermal-induced nocifensive behavior. Pin, Dia, mixture of Gly + Pin, mixture of Gly + Dia or solvent was injected into the rat hind paw in 50 μl. 8 min later, the injected hind paw was subjected to radiant heat from underneath the glass floor with a high-intensity lamp bulb, and paw withdrawal latency was measured and presented. n = 10-13. *p < 0.05, compared with solvent group; #p < 0.05, compared with Pin group. (C) Effects of K_ATP _channel openers on mechanical-induced nocifensive behavior. Pin, Dia, mixture of Gly + Pin, mixture of Gly + Dia or solvent was injected into the rat hind paw in 50 μl. 8 min later, paw withdrawal thresholds (g) were measured using calibrated von Frey filaments applied to the plantar surface of the injected paw. n = 11-12. *p < 0.05, compared with solvent group; #p < 0.05, compared with Pin or Dia groups.

We tested effects of the K_ATP _activators on BK-induced nocifensive behavior in rats. We evaluated nocifensive behavior (time spent in licking, biting and flinching the affected paw) following the hind paw injection of 50 μl of saline containing the relevant compounds. Consistent with our earlier study [20], intraplantar injection of BK (200 μM) into the rat hind paw produced strong nocifensive behavior (quantified within first 30 min after injection; BK, 121 ± 5.7 s, n = 8), which was not observed in rats injected with solvent (0.5% DMSO in saline. 11 ± 7.7 s, n = 8), with the K_ATP _channel activators diazoxide (Dia, 100 μM, 3.8 ± 3.2 s, n = 9) and pinacidil (Pin, 10 μM, 3.6 ± 2.6 s, n = 9), or with the channel blocker glyburide (Gly, 10 μM, 11 ± 8.1 s, n = 9) (Figure 1A). Co-application of the K_ATP _activators with BK (following a pre-application of the activators; see Methods for details.) greatly reduced the BK-induced nociceptive behavior. As demonstrated in Figure 1A, diazoxide (100 μM) and pinacidil (10 μM) reduced the BK-induced pain behavior by approximately half (Dia + BK, 59 ± 8.0 s, n = 8; Pin + BK, 49 ± 5.0 s, n = 8). The solvent control for diazoxide and pinacidil did not affect BK-induced nociceptive effects (DMSO, 0.5%, 120 ± 7.3 s, n = 8; Figure 1A). To test if the antinociceptive effects of the K_ATP _activators could be relieved by blocking K_ATP_, glyburide (10 μM) was applied together with diazoxide or pinacidil. Indeed, glyburide greatly reduced the antinociceptive effects of diazoxide and pinacidil (Figure 1A, Gly + Dia + BK, 104 ± 12.4 s, n = 8; Gly + Pin + BK, 85 ± 10.8 s, n = 8). The experiments presented in Figure 1A were repeated at least three times, and similar qualitative conclusions were reached. Thus activation of K_ATP _effectively reduced BK-induced spontaneous pain behavior.

To confirm that the observed effects of the K_ATP _activators were the results of direct actions on the nociceptors rather than some indirect systemic effects of the K_ATP _activators, two extra control experiments were performed. First, to exclude the possibility that the decreased behavioral responses to BK was the result of a systemic effect of the K_ATP _activators (i.e. lowered blood pressure), pinacidil was injected into the contralateral paw (left) of the paw (right) injected with BK, and the BK-induced behavioral responses were compared with those when pinacidil was co-injected with BK in the same paw (right). As shown in Figure S1A (Additional file 1, Figure S1A), contralateral paw injection of pinacidil did not affect BK-induced behavioral responses. The second control experiments were performed to exclude the possibility that the observed anti-nociceptive effects of K_ATP _activators were the results of the vasodilatation, since the primary effect of pinacidil is a vasodilator. The increase in arteriolar blood flow may potentially affect the behavioral outcome. Although apparent vasodilatation was not observed upon injection of K_ATP _activators visually, a control vasodilator, phentolamine, was used as a control vasodilator to be tested for its effect on nociceptive behavior. Clearly, as shown in Figure S1B (Additional file 1, Figure S1B), phentolamine did not affect the BK-induced behavioral responses.

### The K_ATP _channel activators antagonize thermal pain behavior

We used the Hargreaves test [34], in which the paw is heated by a radiant heat source, to study the effects of the K_ATP _activators on thermal nociception. In this case, the latency to paw licking or withdrawal was measured in the absence or presence of K_ATP _activators and blockers. Thermal nociceptive behavior was studied 8 min after the intraplantar injection of 50 μl of one of the following solutions: solvent alone, the K_ATP _channel activators, and the activators plus blockers. As demonstrated in Figure 1B, compared with the solvent control, both diazoxide (100 μM) and pinacidil (10 μM) significantly increased the time latencies for thermal nociceptive behavior (Solvent, 23 ± 2.8 s, n = 12; Pin, 36 ± 2.9 s, n = 12; Dia 35 ± 3.0 s, n = 13). Pre-injection of glyburide together with the K_ATP _activators reversed the anti-thermal nociceptive effects of the K_ATP _channel activators, although the effects of glyburide on diazoxide did not reach statistical significance (Figure 1B, Gly + Pin, 26 ± 1.7 s, n = 12; Gly + Dia, 31 ± 4.1 s, n = 10). Glyburide alone did not affect thermal nociceptive behavior (26 ± 2.5 s, n = 13). These results show that activation of K_ATP _can reduce the sensitivity to noxious heat stimuli.

### The K_ATP _channel activators antagonize the nociceptive response to mechanical stimuli

We used von Frey filaments to test withdrawal thresholds for mechanical stimuli applied to the hind paw of rats. The solvent control or K_ATP _activators (50 μl) were injected into the plantar of the rat hind paw, and the response to the mechanical stimuli was measured 8 min later. The K_ATP _activators significantly increased the threshold for nocifensive withdrawal of the hind paw in response to mechanical stimuli (Figure 1C, Solvent, 23 ± 3.8 s, n = 12; Pin, 41 ± 6.5 s, n = 10; Dia, 44 ± 5.8 s, n = 12). Pre-injection of glyburide together with the K_ATP _activators reversed the effects of the K_ATP _channel activators (Figure 1C, Gly + Pin, 26 ± 4.9 s, n = 12; Dia + Glib, 23 ± 4.2 s, n = 11). Glyburide alone did not affect mechanical nociceptive behavior (25 ± 5.9 s, n = 11). These results show that activation of K_ATP _can reduce the sensitivity to noxious mechanical stimuli.

### Effects of the K_ATP _activators on the increased excitability of DRG neurons induced by BK

In our earlier study, we showed that BK can depolarize the membrane potential and increase the firing frequency of DRG neurons by inhibiting M-type K^+ ^currents and activating Ca^2+ ^activated Cl^- ^currents [20]. We examined whether activation of K_ATP _could affect the increased excitability of DRG neurons induced by BK.

**Figure 2:**
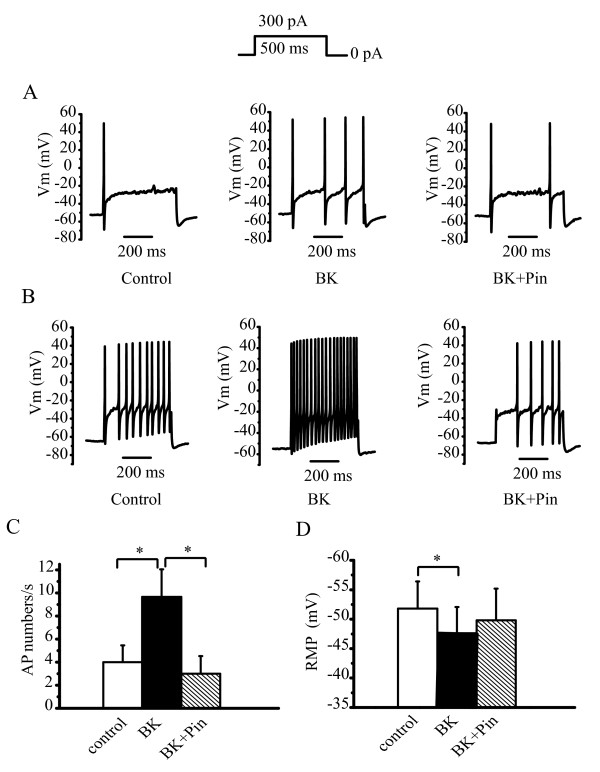
**K_ATP _opener reverses BK-induced hyperexcitability of DRG neurons**. Current clamp recordings from rat DRG neurons; action potentials were elicited by 300 pA current injection for 500 ms period. Either one (A) or multiple (B) action potentials were induced by the depolarization, and the firing frequency was increased by BK (200 nM), which was reversed by pinacidil (Pin, 10 μM). (C) Numbers of action potentials/s (AP) in the absence or presence of BK, pinacidil and BK plus pinacidil. n = 35, **p < 0.01. (D) Resting membrane potentials (RMP) of DRG neurons and the effects of BK, pinacidil and BK plus pinacidil. n = 35, *p < 0.05.

In control neurons under current-clamp conditions (amphotericin B perforated patch recording), the injection of 300 pA depolarizing current elicited either one action potential (AP; Figure 2A) or multiple APs (Figure 2B) in a total of 49 recordings from DRG neurons. There were also 8 neurons that did not produce any firing upon injection of 300 pA (larger depolarization currents were applied on these neurons). In 35 of the 57 neurons, the AP firing frequency was markedly increased by 200 nM BK from 3.7 ± 0.85 AP/s to 8.2 ± 1.43 AP/s (n = 35, p < 0.01; Figure 2C). Pinacidil (10 μM) reversed the BK-induced increased firing (Figure 2C, BK + Pin, AP/s 2.75 ± 0.76, n = 35, p < 0.01). BK also substantially depolarized the resting membrane potential (RMP) from -50.2 ± 3.32 mV to -42.8 ± 3.49 mV (Figure 2D, n = 35, p < 0.05), and this effect was reversed by pinacidil (Figure 2D, RMP -52.9 ± 3.35 mV, n = 35, p < 0.05).

**Figure 3:**
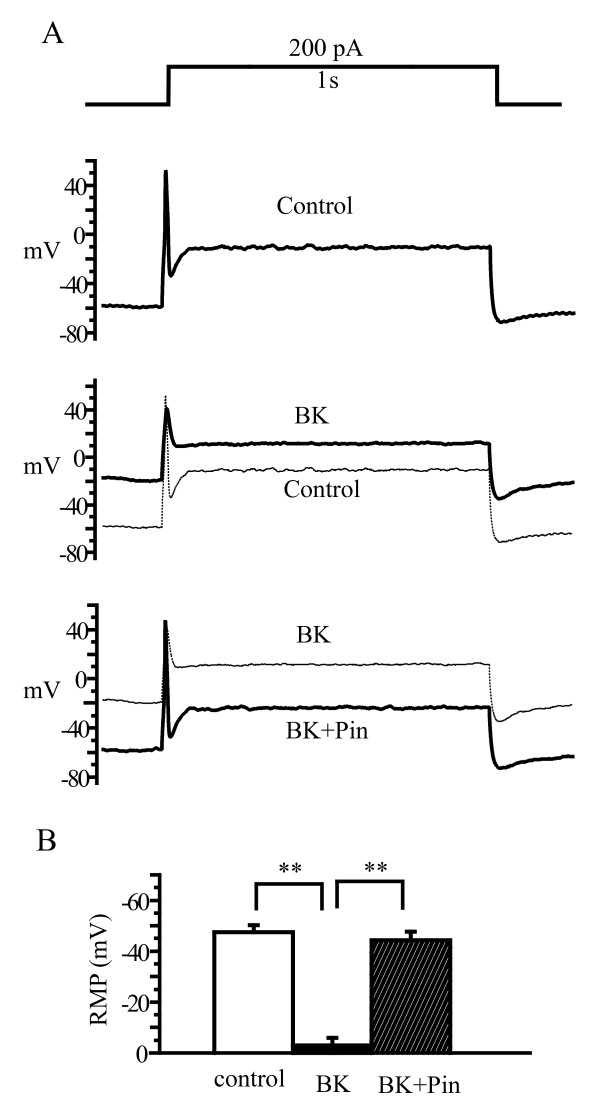
**K_ATP _opener reverses BK-induced marked depolarization**. (A) Representative recordings showing BK-induced marked membrane depolarization without changes in action potential number; pinacidil completely reversed the BK-induced depolarization with no effects on firing frequency of action potential. (B) Summary data for A. n = 7, **p < 0.01.

A subset of DRG neurons responded to BK stimulation differently from those neurons shown in Figure 2. In these neurons, as shown in Figure 3A, the application of BK did not increase the firing frequency of the neurons, but depolarized the membrane substantially. In fact, the membrane was completely depolarized in these neurons (Figure 3B, RMP -47.5 ± 2.81, n = 7 for control, -3.1 ± 2.94, n = 7 for BK, p < 0.05). Pinacidil, when applied in the presence of BK, totally reversed this BK-induced depolarization (Figure 3B, RMP -44.3 ± 3.28 n = 7 for BK + Pin, p < 0.05 compared with BK). Taken together, the above results suggest that activation of K_ATP _could counteract the BK-induced hyperexcitability of DRG neurons.

### Effects of K_ATP _activators and blockers on currents recorded from rat DRG neurons

**Figure 4:**
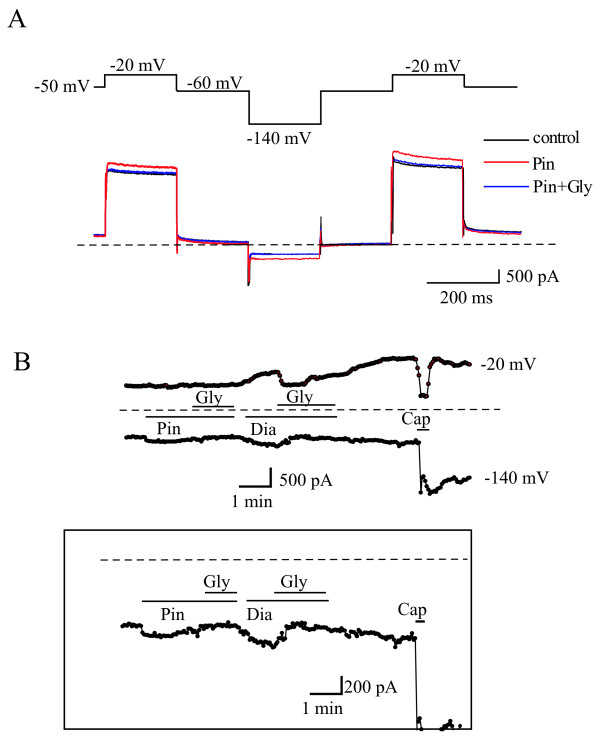
**K_ATP _opener activates glyburide-sensitive currents in DRG neurons**. (A) Representative current traces recorded from a small diameter DRG neuron using the voltage protocol shown at the top. Perforated whole-cell patch clamp was used. Effects of pinacidil (Pin) and pinacidil (Pin) plus glyburide (Gly) were indicated. (B) The time course of currents recorded at -20 mV and -140 mV from a small diameter neuron. Effects of K_ATP _openers and blocker were indicated.

We then designed a voltage protocol to test the effects of K_ATP _activators and blockers on currents recorded from adult rat DRG neurons. The voltage protocol used is shown at the top of Figure 4A. The protocol was designed to serve three major purposes: 1) to record both K_ATP _and M-type K^+ ^currents because M-type currents are believed to be the major K^+ ^currents that control general neuronal excitability [18,19] and particularly DRG excitability [3,20]; 2) to record currents around the resting membrane potential level (-50~ -60 mV) and at the potential for an optimal manifestation of K_ATP _currents (-140 mV); 3) to minimize the activation of other depolarization-activated K^+ ^currents. Thus, M-type currents can be observed at -20 mV (activating currents) and at -50 mV (deactivating tail currents), and K_ATP _currents can be observed at -140 mV as well as at around -60 mV.

The currents recorded using the protocol described above are shown in Figure 4. The dotted line in Figure 4A indicates the zero current level. Substantial basal outward currents were observed at -20 mV, whereas small inward currents were seen at -140 mV. Small slow deactivating tail currents were observed when the membrane potentials were step changed from -20 mV to -50 mV or -60 mV, and these currents eventually reached the zero current level when the membrane potential was at -60 mV (Figure 4A, control). These characteristics of the basal currents are consistent with the presence of outwardly rectifying K^+ ^currents (K^+ ^reversal potential is calculated to be around -100 mV). The resting membrane potential for this cell should be around -60 mV. Pinacidil (10 μM) evoked a small increase of both the outward currents at -20 mV and the inward currents at -140 mV, and these effects were blocked by glyburide (10 μM). Neither pinacidil nor glyburide significantly affected the currents at -50 mV or -60 mV (Figure 4A). Figure 4B shows the time course of the currents recorded at -20 mV and - 140 mV. Pinacidil and diazoxide slightly increased the inward current at -140 mV and the outward currents at -20 mV, and these effects were also reversed by glyburide, consisted with the increased currents being K_ATP _currents. To demonstrate the effect of pinacidil and diazoxide more clearly, the current traces from -140 mV were rescaled (Figure 4B, squared box). Capsaicin, an agonist of the TRPV channel, was applied at the end of the experiments and usually evoked a large inward current at both -140 mV and -20 mV (Figure 4B).

**Figure 5:**
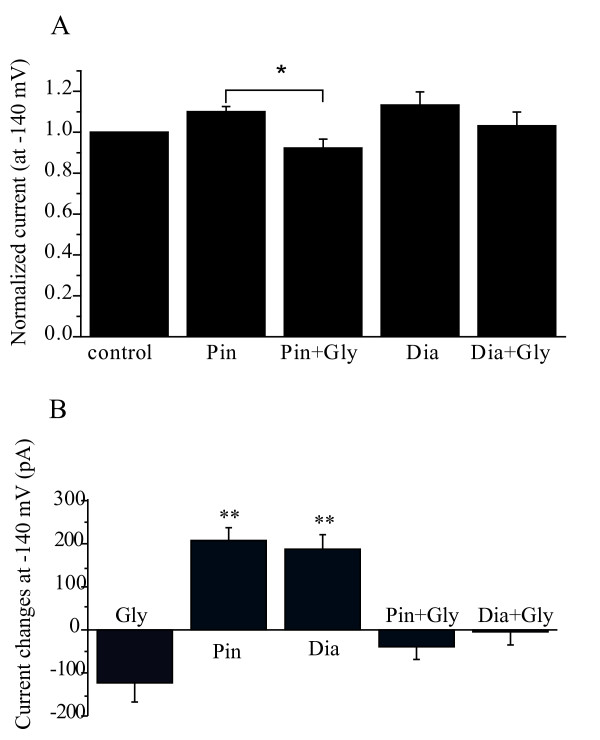
**K_ATP _opener-activated currents in DRG neurons**. (A) Effects of K_ATP _openers and blocker on currents recorded at -140 mV from DRG neurons. n = 12-20, the currents were normalized based on the control basal currents. (B) Absolute increased or reduced currents by K_ATP _openers or blocker recorded at -140 mV. The basal control current levels were taken as zero current level. n = 7-25, **p < 0.05.

The effects of pinacidil and diazoxide are summarized in Figure 5. Figure 5A shows normalized currents recorded at -140 mV before and after the application of K_ATP _channel activators alone or with glyburide. Although both pinacidil and diazoxide increased the inward currents at -140 mV by ~10% and the effects were inhibited by glyburide, the difference did not reach statistical significance (P > 0.05).

The basal currents were large compared with the increased currents by K_ATP _activators (Figure 4). Because we do not know the source of these currents, including these currents in the analysis of the effects of the K_ATP _activators and blocker (Figure 5A) could underestimate the effects of the K_ATP _activators. In addition, not all neurons responded to the K_ATP _activators, but these neurons were included in the analysis shown in Figure 5A. To better assess the actions of the K_ATP _activators and blocker, we re-analyzed the data, and the results are shown in Figure 5B. In this case, only neurons that had a visible response to the K_ATP _activators (31 out of 60) and blocker (6 out of 60) were included for analysis. Furthermore, the basal current levels were taken as a zero current level, and only the currents increased or reduced by the K_ATP _activators and blocker were used for the analysis. Thus, the basal currents were excluded from the analysis. Figure 5B shows that both pinacidil (n = 25) and diazoxide (n = 16) significantly activated currents recorded at -140 mV, and these effects were abolished by glyburide. Of the 60 neurons tested, 23 did not respond to the K_ATP _activators or blocker and were not included in the analysis shown in Figure 5B. These results suggest that the K_ATP _activators activate the K_ATP _currents in a subpopulation of DRG neurons.

## Discussion

In this study, we described the antagonistic effects of the K_ATP _activators, pinacidil and diazoxide, on nocifensive behavior induced by bradykinin, thermal and mechanical stimuli and the bradykinin-induced hyperexcitability of DRG neurons. We also found that the K_ATP _activators can moderately activate K_ATP _in DRG neurons. Because the effects of the K_ATP _activators could be reversed by the K_ATP _blocker glyburide, the direct activation of K_ATP _is the most likely the underlying mechanism. This systematic study clearly demonstrates that activation of K_ATP _could have significant modulatory effects on excitability of the sensory neurons and thus on sensory behavior, such as nociception.

Many studies have provided evidence suggesting that analgesic agents, such as opioid and non-opioid receptor agonists, exert their antinociceptive effects, at least partly, through activation of K_ATP _[9]. In these studies, the involvement of K_ATP _was suggested from the fact that the antinociceptive effects of these analgesic agents could be inhibited by a K_ATP _blocker. Thus, K_ATP _activators are expected to show antinociceptive effects. However, conflicting results were obtained when K_ATP _activators were tested against different pain models. Furthermore, even in the studies that did observe antinociceptive effects, the effects were attributed to the K_ATP_-mediated activation of opioid receptors [9]. In these studies, K_ATP _activators were given via intracerebroventricular (i.c.v.), intrathecal (i.t.) or epidural injection. Thus, the direct effects of K_ATP _on the excitability of sensory neurons could not be determined from these studies. A few studies have also observed the effects of K_ATP _activators on peripheral nociception. In these studies, K_ATP _activators were tested against the hyperalgesia caused by carrageenan or prostaglandin E(2) or in a formalin pain model. The activators reduced pain behavior in these studies [31-33]. In the first part of our present study, we studied the effects of two K_ATP _activators, pinacidil and diazoxide, on three different pain models, and the results unequivocally demonstrate that activation of K_ATP _antagonizes the nocifensive behaviors induced by bradykinin and thermal and mechanical noxious stimuli. Bradykinin is a well-known inflammatory mediator and one of the most potent endogenous pain-inducing substances [35,36]. In our earlier study, we attributed BK-induced spontaneous pain to the inhibition of M channel K^+ ^currents and the activation of Ca^2+^-activated Cl^- ^currents in DRG neurons [20]. BK can also induce hyperalgesia and allodynia [36,37], possibly mediated by sensitization of TRPV1 [38] and TRPA1 [39] through the activation of PKCε [40]. Activation of M channels by the specific channel activator retigabine antagonizes BK-induced pain behavior, indicating an important role for M-type K^+ ^channels in the control of sensory neuronal excitability [20]. On the other hand, thermal and mechanical nociception are mediated by TRPV channels [2,41-44]. Clearly these different nociceptive stimuli employ different mechanisms for inducing nociception, but they should all initiate the nociceptive signals by increasing the excitability of nociceptors. The non-discriminating effects of K_ATP _activators in antagonizing the nocifensive behaviors induced by different stimuli indicate the functional presence of K_ATP _in all the nociceptors mediating bradykinin-induced and thermal and mechanical nociception. On the other hand, the K_ATP _blocker glyburide did not affect any of the three nocifensive behaviors in the absence of K_ATP _activators. This suggests that K_ATP _does not necessarily play a role in the nociception induced by these stimuli, merely that the existence of these channels provides a means of reducing hyperexcitability when K_ATP _activity is enhanced.

Effects of K_ATP _activators on excitability of DRG neurons are in agreement with the results from our nocifensive behavioral study. Thus, pinacidil reversed the hyperexcitability induced by BK in DRG neurons. In addition, pinacidil also reversed the membrane depolarization induced by BK. These effects of pinacidil are very efficient and striking, and in all cases, the effects of BK were completely reversed by pinacidil (Figure 2, 3 and 4). On the other hand, neither the K_ATP _activators nor the blocker *per se *affected the firing frequency or resting membrane potentials of DRG neurons in the absence of BK. Thus, as we observed in the nocifensive behavior study, the effects of K_ATP _can only be manifested when nociception/DRG hyperexcitability/depolarization were initiated by nociceptive stimuli. An increase in the inward currents and/or a decrease in the outward currents is the first step in the activation of nociceptors, which subsequently depolarizes the membrane and increases the firing of APs. This scenario may provide a setting for the manifestation of K_ATP _function. As demonstrated in a previous report, BK increases the excitability of DRG neurons through the inhibition of M-type currents and the activation of Ca^2+^-activated Cl^- ^currents [20]. M-type K^+ ^channels might be the dominate contributor to the resting K^+ ^conductance [45] in neurons, thus masking the contribution of other K^+ ^channels, such as K_ATP_. The reduction of M-type K^+ ^currents would allow the function of other K^+ ^channels to be unmasked. In addition, membrane depolarization (Ca^2+ ^influx and inward currents through Cl^- ^currents) would increase the driving force for outward K_ATP _currents. The combination of these two changes could explain the observed effects of K_ATP _activators under nociceptor activation. This possibility may not be limited to the explanation of K_ATP _activators antagonizing BK effects. In fact, with regard to the modulation of M-type currents and membrane depolarization, other nociceptive stimuli may result in same molecular environment as BK. The core of the BK mechanism is an increase in intracellular Ca^2+ ^[20], which should be shared by many other stimuli which activate TRPV channels.

In the present study, K_ATP _only induced small glyburide-sensitive currents in a subpopulation of rat DRG neurons. This modest effect of K_ATP _activators indicates that K_ATP _function is not subject to modulation under resting conditions. Furthermore, lack of significant effects of glyburide on the basal currents of DRG neurons further implies that K_ATP _does not contribute substantially to the resting membrane conductance of DRG neurons. This is in line with the observations from our nocifensive and excitability experiments. A systematic analysis of K_ATP _distribution in subgroup DRG neurons is lacking. Population of the small size DRG neurons are believed to be the nociceptors. Clearly, these small DRG neurons have functional K_ATP _[15]. However, the activity of the K_ATP _in the small DRG neurons seems lower than that of the K_ATP _in the large DRG neurons, and furthermore, these K_ATP _in two sizes of DRG neurons may contributed differently to the development of the neuronpathic pain [15]. The small nociceptive DRG neurons are polymodal and are categorized into two major classes based on peripheral and central target fields, trophic factor dependence, and biochemical properties [46]. These two classes of nociceptors, nonoverlapping populations of trkA positive (tyrosine kinase A) peptidgeric and isolectin-B4 (IB4) positive neurons may have different mechanisms in controlling the excitability and have different responsiveness to different stimuli. The expression of K_ATP _channels in peptidergic nociceptors (CGRP positive) has been shown [17]. However, a systematic functional and expression study of K_ATP _in defined subpopulation of DRG neurons will help to understand the role of the K_ATP _in nociception further.

It seems that K_ATP_-like currents can be readily recorded at a single channel level from cell-attached or isolated inside-out patches [13-15,17,20]. Although all K_ATP _channel subunits (Kir6.1 and Kir6.2) and SUR receptors (SUR1 and SUR2) can be found in DRG neurons at mRNA level, pharmacological, western blots and immunohistochemistry studies suggest that the single channel activity of K_ATP _in DRG neuron may be the results of heteromeric Kir6.2/SUR1 complex [17]. The reports for whole-cell K_ATP_-like currents in DRG neurons are scarce; only two published papers have reported K_ATP_-like whole cell currents from DRG neurons. In one of these works, K_ATP _activator- and blocker-sensitive outwardly rectifying currents were recorded [8]. This study also reported that the currents conveyed by K_ATP _account for only 10-25% of the total potassium currents recorded [8]. In the present study, we recorded a 10% increase over the basal currents in response to K_ATP _activators (Figure 5A). In another report, the K_ATP _activator diazoxide activated currents in only a subpopulation of DRG neurons [11]. However, the diazoxide-activated currents were not subjected to testing for inhibition by a K_ATP _blocker [11]. Further experiments are needed to define the pharmacological and biophysical properties of the whole-cell K_ATP_-like currents in DRG neurons. Taken together, our work and that of other groups suggest that K_ATP _is not a dominant contributor to conductance under resting conditions in DRG neurons.

In summary, our behavioral and electrophysiological results support the notion that functional K_ATP _channels are present in nociceptors. K_ATP _opening could antagonize the nociceptive responses evoked by noxious stimuli by dampening the hyperexcitability of the nociceptors. On the other hand, K_ATP _may not contribute significantly to the resting membrane conductance of the DRG neurons. Nevertheless, this should not dampen enthusiasm for further investigation of K_ATP _activators as potential candidates for antinociceptive measures. In light of the findings that analgesic compounds, such as morphine, may exert antinociceptive effects through activation of K_ATP _[16,27], direct activators of K_ATP_, such as pinacidil and diazoxide, are certainly worthy of further investigation for their potential analgesic actions. In this regard, our behavioral results concerning these compounds provide a solid foundation for future studies. The characteristics of molecular compositions, pharmacological modulation and biophysical properties of the K_ATP _in DRG neurons need to be further investigated in order to find whether the K_ATP _in DRG neurons can be specifically modulated by the channel activator to relieve the pain induced by different stimuli.

## Methods

### Behavioral studies

Male Sprague-Dawley rats (180-220 g) were randomly grouped and allowed to acclimatize for at least 20 min to the environment prior to the experiment. Behavioral studies were conducted in three separate experimental settings: (1) Bradykinin-induced acute spontaneous pain. The right hind paw of the animal received an intraplantar injection (50 μl) of BK (200 μM, 10 nmol/site) or solvent (saline) and the nocifensive responses (licking, biting, lifting and flinching) were recorded using a video camera for 30 min. The videos were analyzed by an observer unaware of the treatment allocations. To study the effects of activation of K_ATP _channels on bradykinin-induced nociceptive behavior, one group of animals was pre-injected with the K_ATP _activators. After 5 min, bradykinin(BK) was co-injected with the activators into the same site of the hind paw. Control animals were injected with solvent (0.5% DMSO in saline) instead of the K_ATP _activators. In a second experimental group, the K_ATP _blocker was co-applied with the K_ATP _activators. Drugs were diluted in saline (pH 7.4) from stock solutions (dissolved in DMSO) and applied at a volume of 50 μl at the following concentrations: diazoxide, 100 μM; pinacidil, 10 μM; glyburide, 10 μM. (2) Mechanical withdrawal thresholds were measured using calibrated von Frey filaments (a set of monofilaments made from nylon filaments of varying diameter) (North Coast Medical, Inc. Morgan Hill, CA) applied to the plantar surface of the paw. Testing was initiated with an Evaluator Size 5.07 (10 g). If the animal withdrew the paw, the next weaker hair was applied. In the case of no withdrawal, the next stronger hair was applied [47]. The cut-off was Evaluator Size 6.10 (100 g). (3) To test for thermal hyperalgesia, radiant heat was applied to the plantar surface of a hind paw from underneath a glass floor using a ray of light from a high-intensity lamp bulb. The paw withdrawal latency was recorded automatically when the paw was withdrawn from the light (TaiMeng Technology Co., Ltd. Chengdu).

### Rat DRG cell culture

The dorsal root ganglia were extracted from all spinal levels of 32 adult male Sprague Dawley rats, and the neurons were dissociated as previously described [20]. Briefly, the rats were anesthetized with an intraperitoneal (*i.p.*) injection of pelltobarbitalum natricum (10-20 mg/kg) and then sacrificed. The ganglia were cut into pieces, transferred into collagenase solution (1 mg/ml) and incubated for 50 min at 37°C. The ganglia were then placed into a trypsin solution (2.5 mg/ml) for 20 min at 37°C. The digested fragments were then rinsed with 2 ml DMEM plus 10% fetal bovine serum three times, centrifuged and dissociated by trituration. The ganglia were plated onto glass coverslips pre-coated with poly-D-lysine and incubated at 37°C. After the neurons had attached to the coverslips, the cell culture medium was changed to Neurobasal plus B27 supplement (Invitrogen). Neurons were used within 24 h of isolation. The diameter of the DRG neuron was measured using a calibrated micrometer mounted in the eyepiece of the microscope.

### Electrophysiology

Patch electrodes were pulled from borosilicate glass and fire-polished to a final resistance of 1-2 MΩ when filled with internal solution. An Axon 700B (Axon Instruments) patch clamp amplifier was used for voltage and current clamp experiments. All recordings were performed using the amphotericin B (250 μg/ml, Sigma) perforated patch technique. The internal pipette solution contained (in mM) 150 KCl, 5 MgCl_2_, 10 HEPES, pH 7.4. The external solution contained (in mM) 160 NaCl, 2.5 KCl, 5 CaCl_2_, 1 MgCl_2_, 10 HEPES, and 8 glucose, pH 7.4. A low-profile perfusion chamber fed by a gravity perfusion system was used for solution exchange (2 ml/min, bath exchange time of ~15 s).

### Statistics

All data are given as mean ± SEM. Differences between groups were assessed by Student's *t*-test or one-way ANOVA followed by Bonferroni's post-hoc test. The differences were considered significant at p ≤ 0.05.

### Chemicals

Diazoxide; pinacidil; glyburide and capsaicin were obtained from Sigma.

The use of animals in this study was approved by the Animal Care and Ethical Committee of Hebei Medical University, (Shijiazhuang, China) under the IASP guidelines for animal use.

## Competing interests

The authors declare that they have no competing interests.

## Authors' contributions

XD participated in the design of the study and carried out the electrophysiological studies, participated in the behavioral studies. CW carried out the behavioral studies, participated in the electrophysiological studies. HZ conceived of the study, participated in its design and coordination, and drafted the manuscript. All authors read and approved the final manuscript.

## Supplementary Material

Additional file 1 **Fig S1. The anti-nociceptive effects of pinacidil were not indirect results from the effects on targets other than nociceptors**. A, Pinacidil 10 μM was either injected contralaterally or ipsilaterally with BK. BK (200 μM) was injected into the right hind paw of the rats. For the contralateral injection, pinacidil was first injected into the left hind paw of the rat, and 5 min later, pinacidil was again injected in the left hind paw and at the same time, BK was injected into the right hind paw of the rats. The nocifensive behavior (the time the animals spent licking, biting and flinching the injected paw during 30 min) in the right hind paw was counted. For the ipsilateral injection, pinacidil was injected first into the right hind paw of the rats, and 5 min later, pinacidil plus BK were injected in the right hind paw again. The nocifensive behavior in the right hind paw was counted. B, Either pinacidil or phentilamine (100 μM) was injected ipsilaterally with BK and the nocifensive behavior was analyzed with the same protocol described above and in the Methods. **p < 0.01, compared with the contralateral (A), and compared with the control (B), n = 8-12. Click here for file
