# Supplementary figures and images for: Activation of ATP-sensitive potassium channels antagonize nociceptive behavior and hyperexcitability of DRG neurons from rats
Source: Mol Pain. 2011 May 14;7:35. doi: 10.1186/1744-8069-7-35 (PMC3113320; doi:10.1186/1744-8069-7-35)

A

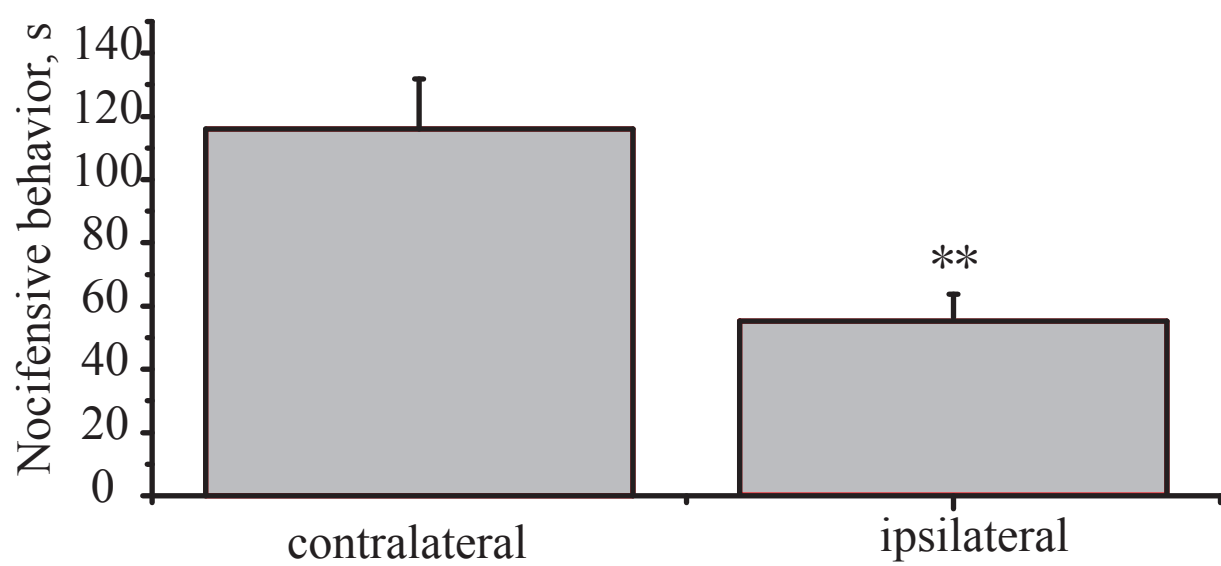

B

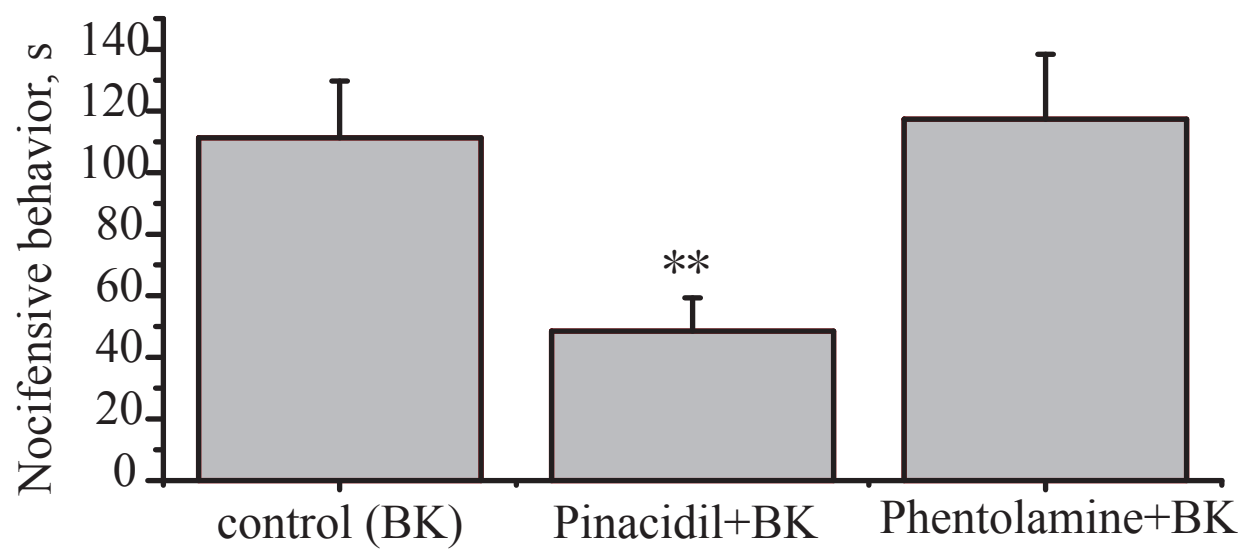

Supplement: Additional file 1 — Fig S1. The anti-nociceptive effects of pinacidil were not indirect results from the effects on targets other than nociceptors. A, Pinacidil 10 μM was either injected contralaterally or ipsilaterally with BK. BK (200 μM) was injected into the right hind paw of the rats. For the contralateral injection, pinacidil was first injected into the left hind paw of the rat, and 5 min later, pinacidil was again injected in the left hind paw and at the same time, BK was injected into the right hind paw of the rats. The nocifensive behavior (the time the animals spent licking, biting and flinching the injected paw during 30 min) in the right hind paw was counted. For the ipsilateral injection, pinacidil was injected first into the right hind paw of the rats, and 5 min later, pinacidil plus BK were injected in the right hind paw again. The nocifensive behavior in the right hind paw was counted. B, Either pinacidil or phentilamine (100 μM) was injected ipsilaterally with BK and the nocifensive behavior was analyzed with the same protocol described above and in the Methods. **p < 0.01, compared with the contralateral (A), and compared with the control (B), n = 8-12. [file 1744-8069-7-35-S1.PDF]
